# Estimation of leaf nutrition status in degraded vegetation based on field survey and hyperspectral data

**DOI:** 10.1038/s41598-020-61294-7

**Published:** 2020-03-09

**Authors:** Yu Peng, Mei Zhang, Ziyan Xu, Tingting Yang, Yali Su, Tao Zhou, Huiting Wang, Yue Wang, Yongyi Lin

**Affiliations:** 1 College of Life & Environmental Sciences, Minzu University of China, Haidian District, Beijing, 100081 China; 2 State Key Laboratory of Earth Surface Processes and Resource Ecology, Beijing Normal University, Beijing, 100875 China

**Keywords:** Ecophysiology, Restoration ecology

## Abstract

Timely monitoring of global plant biogeochemical processes demands fast and highly accurate estimation of plant nutrition status, which is often estimated based on hyperspectral data. However, few such studies have been conducted on degraded vegetation. In this study, complete combinations of either original reflectance or first-order derivative spectra have been developed to quantify leaf nitrogen (N), phosphorus (P), and potassium (K) contents of tree, shrub, and grass species using hyperspectral datasets from light, moderate, and severely degraded vegetation sites in Helin County, China. Leaf N, P, and K contents were correlated to identify suitable combinations. The most effective combinations were those of reflectance difference (Dij), normalized differences (ND), first-order derivative (FD), and first-order derivative difference (FD(D)). Linear regression analysis was used to further optimize sensitive band-based combinations, which were compared with 43 frequently used empirical spectral indices. The proposed hyperspectral indices were shown to effectively quantify leaf N, P, and K content (R2 > 0.5, p < 0.05), confirming that hyperspectral data can be potentially used for fine scale monitoring of degraded vegetation.

## Introduction

There is an increasing need for a method to monitor global plant biogeochemical processes, requiring rapid and accurate estimation of plant nutrition status at multiple scales^1–3^. Remote sensing, especially hyperspectral remote sensing, is a suitable method for this purpose^3,4^. Leaf nitrogen (N), phosphorus (P), and potassium (K) contents are powerful indicators of plant nutrition status^5–8^. Around 50–75% of total plant nitrogen is allocated to chloroplasts to participate in photosynthesis^8^. Since leaf Rubisco activity is highly correlated with leaf N content^6^, strong correlations exist between leaf chlorophyll and N content^5,7^. Equally, N deficiency can directly affect vegetation productivity and delay growth due to community competition. Sufficient supply of N contributes to the ecological restoration of degraded vegetation.

Extensive studies have been conducted to estimate N content through chlorophyll-based spectral indices^9–12^, using techniques such as selecting sensitive wavelengths related to N, or by acquiring spectral reflectance data from multiple sensors with a variety of spatial resolutions^13^. Leaf N has been found to relate to leaf or canopy reflectance more closely in the green wavelengths than in the red-edge or NIR regions^14^. However, when comparing correlation coefficient (R^2^) values, the 17 most sensitive bands selected from the total 85 lie in the visible and NIR spectra (538 nm to 910 nm). These were found to be closely associated with leaf N content^15^. At the same time, the wavelengths most sensitive to leaf N content varied with the spatial scales at which the measurements were made, and with the growth stages of the plants being monitored^15–17^. In summary, approaches that use purely statistical analysis are subject to site-specific problems, and the specific wavelengths selected to estimate plant N status using this method could change from one location to another. Furthermore, most studies have only used a limited number of wavelengths in specific spectral regions to calculate spectral indices, and have not exploited the full spectrum information. Based on this, we suggest that the identification of sensitive wavelengths from the entire range of spectra, rather than limited bands, would be a more precise way to estimate leaf N content.

P is a second key part of plant nutrition, utilized in cell membranes, nucleic acids, and various enzymes, and can also be used to determine a plant’s health status. Major functions of P are related to carbon assimilation, where a deficiency in P will decrease carbon metabolism, prevent the synthesis of various chemical compounds, and ultimately degrade canopy biomass^9^. In order to rapidly monitor plant P status, remote sensing has been employed in biomes such as grasslands and savannas^1,18–20^, crops^21^, and trees^22^. A study of maize leaf P content found that 540, 720, 740, and 850 nm are the most sensitive bands for detection of P in both the vegetation production stage and the flowering stage^21^.

The spectral region which most closely relates to leaf P content has been found to overlay the spectral region which demonstrates water absorbing traits (1000–2500 nm)^19^. Bands which are indicative of leaf P also lie in the region of 580–710 nm, although this varies among different case studies. The confusion between water absorption and sensitivity to sampling sites makes it difficult to identify the most suitable bands for leaf P estimation. In this study, we aimed to select several sensitive bands from the 500 available and develop a complete combination of reflectance and its first-order derivative (FD) from tree, shrub, and grass species in various degraded vegetation sites, with the objective of developing more general hyperspectral indices for the estimation of leaf P content.

Finally, potassium (K) is also a key plant requirement, present mostly as K + ions in vacuoles. K provides regulatory control over processes such as transpiration, starch synthesis, sucrose translocation, respiration, and lipid synthesis^23^. Plants deficient in K exhibit limited growth, metabolism, and stress defense^24^, leading to lower overall biomass and coverage and changes to leaf color. If a K deficiency occurs at the vegetation level, this can accelerate the degradation process. Soils in many broad-acre semiarid areas have become deficient in K, resulting in a decrease of K in the canopy and stem^25,26^.

Remote sensing of leaf N, P, or K contents is a challenging task due to the lack of direct absorption features that can be observed in the spectra. A standard research approach exploits existing correlations between leaf nutrition and biophysical variables such as leaf chlorophyll or area index that shape the reflectance spectra to estimate leaf N, P, or K contents. Remote sensing of plant nutrients has mostly been applied to N^2,27^, and only a limited number of studies have attempted to classify K deficiency, including studies of wheat^27,28^ and rice^29^. In these, the R^2^ for leaf K content with FD at every band from 12 plant species in northern China indicated that the sensitive bands are located at wavelengths of 570, 770, and 1070 nm^22^. A study on apple canopy K content indicated sensitive bands at 706 nm for reflectance (R), 922 nm for FD, 351 nm for 1/R, 359 nm for FD of 1/R, 351 nm for lgR, 922 nm for FD of lg(R), 706 nm for square root of R, and 922 nm for the FD of R’s square root^30^. No studies have assessed the relationship between K-based spectral indices and field measured K content in temperate degraded vegetation. We hypothesize that spectral indices for leaf K content will vary along degradation intensities, with the aim of developing new spectral indices to estimate leaf K content with high accuracy and generality.

The monitoring of degraded vegetation is an important issue for ecological restoration worldwide^31^, since approximately a third of land surface can now be regarded as being degraded to some degree^32^. Because plant nutrition status indicates the overall health of vegetation, and hence its degradation status, such measurements are urgently needed in order to rapidly monitor plant degradation in a nondestructive manner. This will enable restoration and re-vegetation actions where they are required^1–3^. However, there have been relatively few investigations conducted to develop hyperspectral indices for estimating plant nutrition status, despite this being a key physiological parameter for plant status monitoring, and spectral indices correlating to varying degradation intensity is unclear. We therefore aimed to develop suitable indices based on wavelengths from 350 to 1000 nm, and to identify the best indices for estimating leaf N, P, and K contents in temperate degraded vegetation.

## Materials and Methods

### Study area

**Figure 1:**
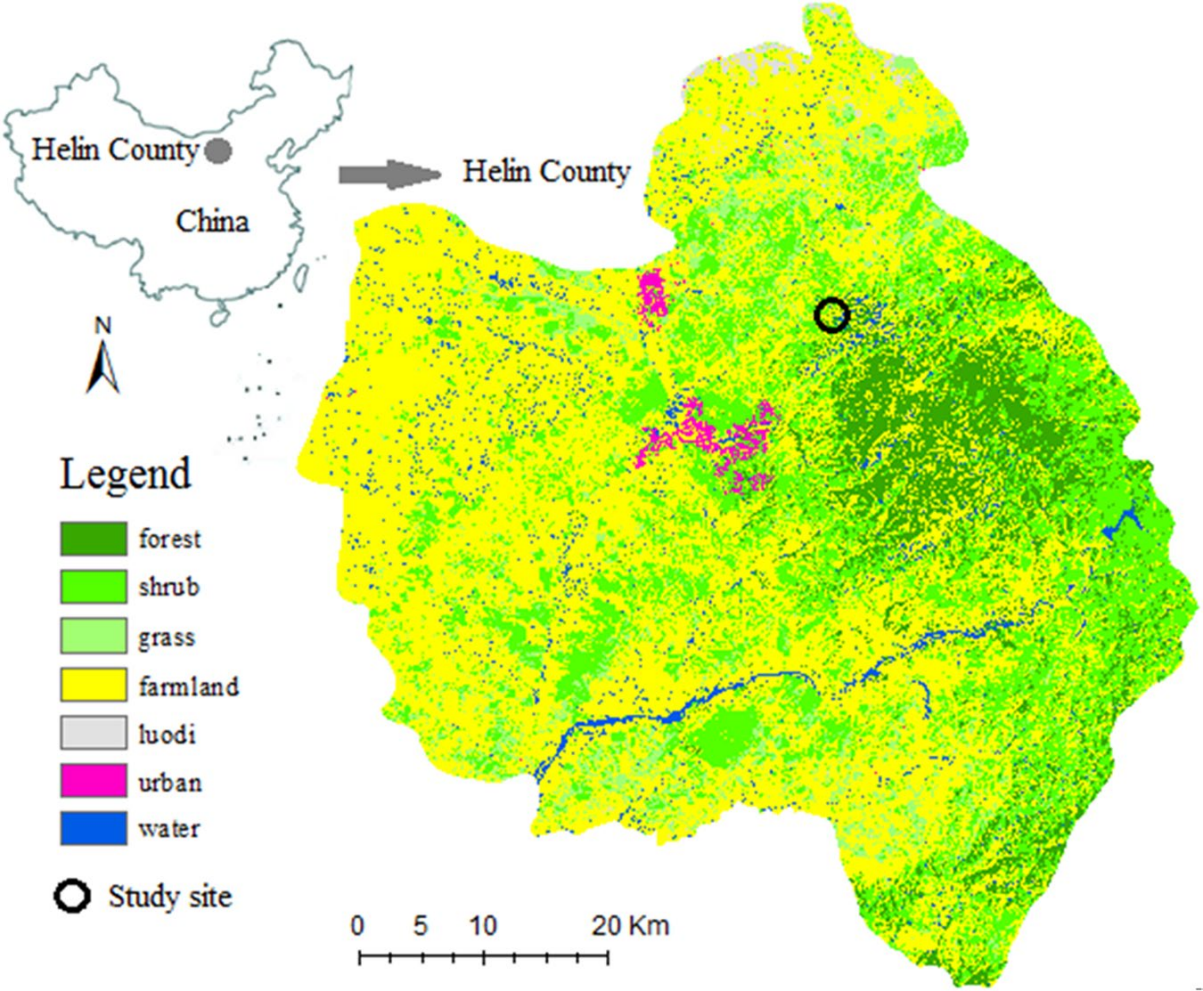
Location and land use distribution of the study area in Helin County, northern China. Map created using ArcGIS 10.2 software (ESRI, Redlands, CA, USA) by the first author.

The study was conducted in Helin County, Inner Mongolia, north China. Helin County is located in the northern agro-pastoral ecotone, composed of flat plains, hills, and mountains in almost equal proportion (Fig. 1). The average elevation of the county is 1176 meters above sea level and the total area is 3401 square kilometers. With a temperate climate alternated by obvious wet and dry seasons, Helin County has annual average temperature of 5.6 °C, averaging −12.8 °C in January and 22.1 °C in July. The average annual precipitation is 417 millimeters, averaging 30 millimeters in January and 103 millimeters in July. The average wind speeds are slightly higher in spring and winter than in the summer and fall seasons. The average relative humidity for the whole year does not show obvious seasonal changes. The semi-arid climate supports sandy vegetations, in which grass and shrubs are predominant.

### Hyperspectral and leaf nutrition data collection

Data were collected *in-situ* in the degraded, temperate sparse-forest grassland (46°58′2.84″N, 17°55′4.34″E) in middle Helin County, China (Fig. 1), where desertification as well as overgrazing and agricultural reclamation and have caused vegetation degradation. The Helin County restoration program ecological experts grade degradation as either light, moderate and severe. Light degraded vegetation is characterised by higher canopy coverage (76%,), species diversity (richness is 32 and Shannon index is 2.36) and soil moisture (relative weight, 24%), followed by moderate (52%, 28 and 1.34, 16%) and severe degraded vegetation (33%, 22 and 0.88, 7%).

Data was collected over 28 days under clear sky conditions between 10:00 and 14:00 local time in July and August in 2012 and 2013. This time is characterized as peak vegetation conditions in the area. Leaf N, P, and K contents and hyperspectral data were recorded for the three different treatments frm the middle leaf samples of eight plants in each six dominant plant species (Setaria viridis, Agropyron cristatum, Salsola collina, Caragana microphylia, Lespedeza davurica, Pinus sylvestnis var.mongolica). Hand–Held ASD portable FieldSpec 2 spectrometer (Analytical Spectral Devices Inc., USA) recorded hyperspectral data. The spectrometer’s spectral range is 325 to 1075 nm, 1 nm bandwidth (www.asdi.com). A leaf clip attaching the fiber optics to the leaves provided reflectance values. Once the leaf reflectance measurements were complete, leaf samples were collected, oven-dried at 70 °C ± 5 °C for 72 h, and dry matter analyzed for N, P, and K. Leaf N was measured by the Kjeldahl method^33^. Leaf P content was measured using the phosphovanadate method^34^. Leaf K content was analyzed using atomic absorption spectroscopy^22^. Results were expressed as mg (N, P, K) g-1 leaf dry matter. 144 original reflectance and leaf nutrition measurement samples were collected (8 plants from 6 species at 3 intensities). 64-pair samples were used to create spectral models, 80-pair samples (for empirical indices) and 30-pair samples (for new indices) were used for validation of leaf nutrition content.

### Reflectances differentiates along degradation intensity

We first investigated the vegetation reflectance at various degradation intensities and estimated the spectral response differentiates. The spectral reflectances of the leaves of dominant plant species were measured at three degradation intensities: light, moderate and severe. We used detrended canonical correspondence analysis (DCCA) to study the spectral response differentiates. DCCA uses two matrixes: a matrix of response variables, which is denoted as Y and often contains the degree of vegetation degradation and a matrix of explanatory variables (e.g. reflectance at each band), which is denoted as X and used to explain the variation in Y, as in regression analysis. In DCCA with detrending by segments and Hill’s scaling, the length of the longest axis provides an estimate of reflectances variation. The unconstrained ordination provides basic overview of the compositional gradients in the data. Canoco software for Windows 4.5^35^ was used for DCCA. If DCCA demonstrated statistical discrepancy between degradation intensities, we then subsequently selected the best performing hyperspectral indices, which had the highest consistency across the three degradation intensities. The indices, which had the highest relation coefficients, were selected as the final best indices to predict leaf N, P, and K contents.

### Development and validation of hyperspectral indices

Due to noise effects in the raw data, the marginal ranges 325–350 nm and 1000–1075 nm were removed from each spectrum. Instead of discrimination analysis for selecting the optimum bands, we chose to concentrate on deriving the complete combination of spectral indices between all channels. The aim of spectral indices is to construct a mathematical combination of spectral band values for enhancing the information content in regard to the parameter under study. Most published indices^36^ are expressed as reflectance or a first-order derivative at a given wavelength, wavelength difference (Dij), ratio (RR), normalized difference (ND), or inverse reflectance differences (ID). Ten common types of indices based on both original reflectance and derivative spectra were used:

1 $${\rm{R}}={\rm{Ri}}$$

2 $${\rm{Dij}}={\rm{Rj}}\,-\,{\rm{Ri}}$$

3 $${\rm{RR}}={\rm{Rj}}/{\rm{Ri}}$$

4 $${\rm{ND}}=({\rm{Rj}}-{\rm{Ri}})/({\rm{Rj}}+{\rm{Ri}})$$

5 $${\rm{ID}}=1/{\rm{Rj}}-1/{\rm{Ri}}$$

6 $${\rm{FD}}={\rm{FD}}({\rm{R}})$$

7 $${\rm{FD}}({\rm{D}})={\rm{FD}}({\rm{Rj}})-{\rm{FD}}({\rm{Ri}})$$

8 $${\rm{FD}}({\rm{RR}})={\rm{FD}}({\rm{Rj}})/{\rm{FD}}({\rm{Ri}})$$

9 $${\rm{FD}}({\rm{ND}})=({\rm{FD}}({\rm{Rj}})-{\rm{FD}}({\rm{Ri}}))/({\rm{FD}}({\rm{Rj}})+{\rm{FD}}({\rm{Ri}}))$$

10 $${\rm{FD}}({\rm{ID}})=1/{\rm{FD}}({\rm{Rj}})-1/{\rm{FD}}({\rm{Ri}})$$

where R is reflectance, FD is first-order derivative spectra and the suffixes (i or j) are wavelength (nm). In the entire 350 to 1000 nm wavelength domain, these indices were evaluated by regression analysis with leaf N, P, and K contents.

In order to determine leaf N, P, and K content, we concluded from visual evaluation that the relationships were linear. This allowed us to calculate the coefficient of determination (R^2^), and the corresponding significance level (p), across the complete combinations (Eq. 1–10) on entire wavelength band from 350 nm to 1000 nm. The optimum combination to represent nutritional content is identified as that with the highest R^2^.

Only few bands remained after identification of R^2^. These few bands in optimum combinations were further filtered through stepwise linear regression analysis. This analysis can reduce the redundant collinear spectral variables to a few non-correlated latent variables, thus avoiding the potential overfitting typical in correlation analyses. The formula of the stepwise regression is:

$$Y={{\rm{\beta }}}_{0}+{{\rm{\beta }}}_{1}{X}_{1}+{{\rm{\beta }}}_{2}{X}_{2}+\ldots +{{\rm{\beta }}}_{k}{X}_{k}$$

Where *Y* is explanatory variable (leaf nutrition content); β0 is regression constant; β1 is the partial regression coefficient of the independent variable *X*1 (one band); β2 is the partial regression coefficient of the independent variable *X*2; βk is the partial regression coefficient of the independent variable *X*k; k is the number of independent variables.

In order to evaluate our developed hyperspectral indices, we have derived 43 frequently used empirical indices from the published literature. We compared the performance of the empirical indices and the newly developed hyperspectral indices by comparing the R^2^ values and their significance levels. The models with the largest R^2^ and highest statistical significance would be regarded as the optimal model. The models were calculated and compared by SPSS 19.0 software.

## Results

### Reflectance response to degradation intensity

**Figure 2:**
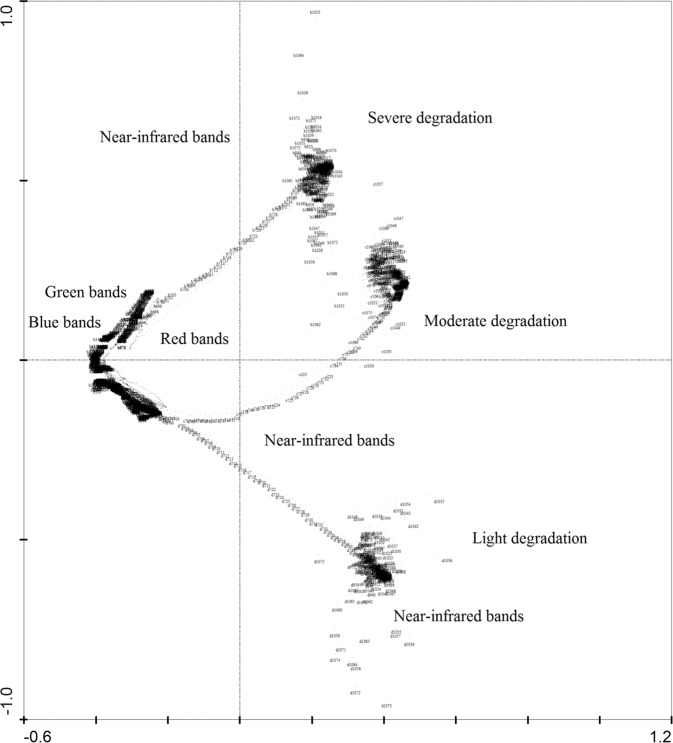
DCCA indicates three clusters of the mean reflectances collected from light, moderate and severe degraded vegetations in Helin County, Inner Mongolia, China. The length of x and y axes (no unit) indicate the relative variation extent in the mean reflectances along degradation intensity.

Cluster distributions of reflectances along degradation intensity are presented in Fig. 2. Differences tended to be more pronounced with greater degradation intensity (Fig. 2). T-tests of the bands at 350 nm optical and 1000 nm NIR regions both indicated significant differences between degradation intensities (p < 0.05), which may be partly explained by the enhancement of vertical leaf distribution in lightly degraded vegetation, which had higher leaf density and canopy cover than severely degraded vegetation^37^. In severely degraded vegetation, there was decreased absorption capacity in the visible and red edge regions of the spectrum alongside decreases in leaf nutrition status, which shifts the reflectance towards the blue end of the spectrum and away from the red in lightly degraded vegetation^38^. Consequently, a cluster of measured reflectance points from lightly degraded vegetation can be separated from those clusters associated with severely degraded vegetation.

### Correlation curves of reflectance-based complete combinations with leaf N, P, and K contents

**Figure 3:**
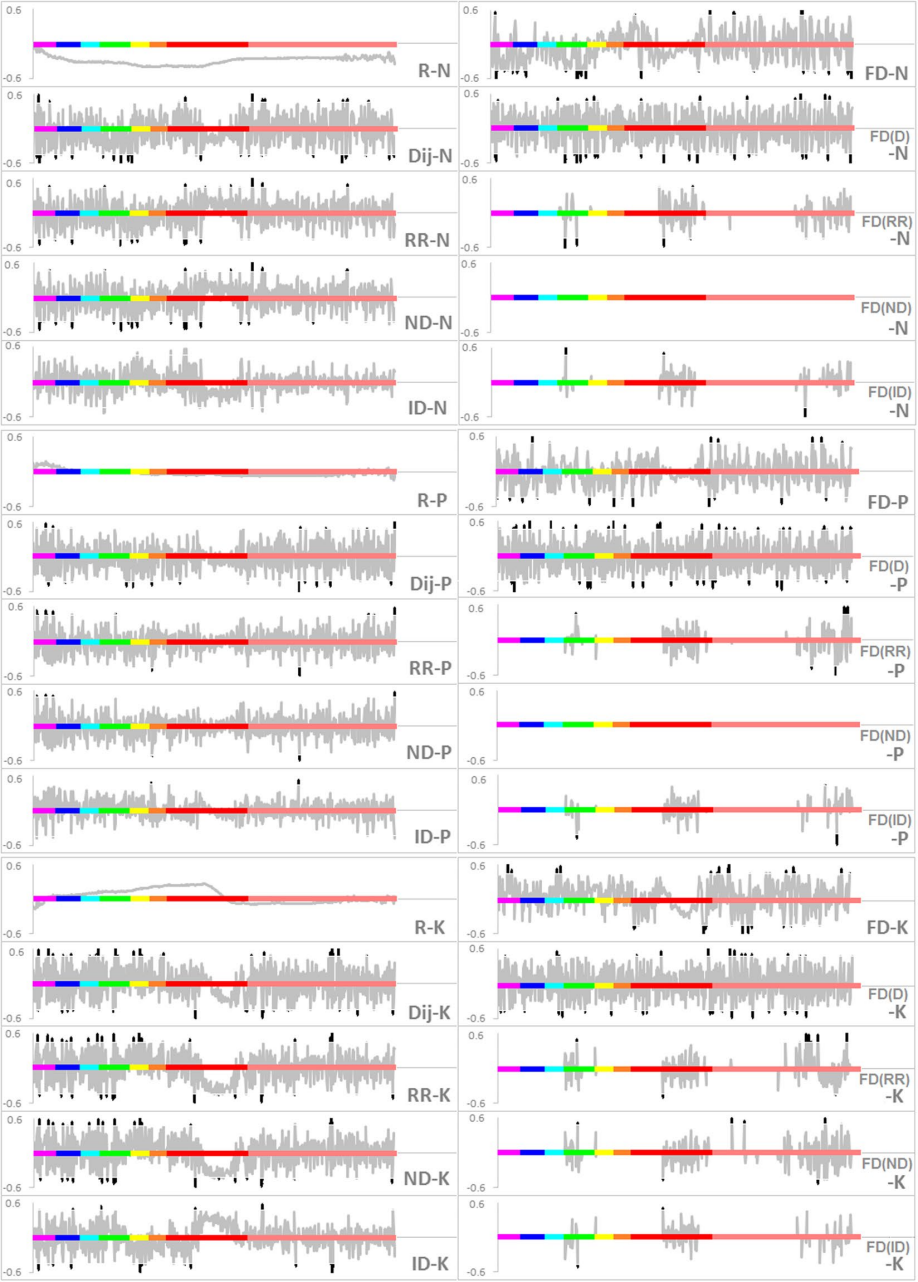
Correlation coefficients of combinations of Ri and Rj and leaf N, P, and K contents, respectively. The X axes indicate the wavelength ranges from violet, blue, cyan, green, yellow, orange, red to near-infrared light (350–1000 nm). The Y axes indicate the correlation coefficient. Curves in grey indicate non-significant coefficients; curves in black indicate significant coefficients (p < 0.05).

Figure 3 presents an indicative subset of the results of the R, Dij, RR, ND, ID, FD, FD(D), FD(RR), FD(ND), and FD(ID) relationships for leaf N, P, and K contents, respectively, showing various combinations of reflectance and plant nutrition content and their correlations. These can be a significant source of information to correlate the physiological parameters under study^36^ which allows optimized selection of effective wavelengths and bandwidths. Considering the various combinations of Ri and Rj, the combinations with the largest number of significant correlation coefficients were Dij, FD(D), RR, and ND for leaf N content; Dij, FD(R), and FD(D) for leaf P content; and Dij, RR, ND, FD, and FD(D) for leaf K content. These were selected as potential combinations for further analysis.

The sensitive bands related to leaf N content were mostly found in the green, green-yellow edge, middle red, and NIR regions of the spectrum. Leaf P content had the fewest significant coefficients in the combinations of reflectance and its FD (Fig. 3). The curves for FD-P and FD(D)-P indicated sensitive bands in the green-yellow edge, red, and NIR regions. The sensitive bands for leaf K content were mainly located in the short wavelength bands, ranging from 360 nm to 450 nm and covering the violet, blue, cyan, green, and yellow regions of the spectrum. FD values did not show a significant relation with leaf nutrient contents.

Dij, ND, RR, FD(D), and FD(R) responded differently to leaf nutrition across wavelengths (Fig. 3). For example, the correlation coefficient between Dij and leaf N content was significant in one band, but non-significant in the neighboring wavebands. Some bands had significant coefficients among several combinations, where they were significant in the Dij, ND, and FD(D) curves. Based on this, the bands with the largest number of significant coefficients among all combinations were determined to be the most sensitive and indicative of plant leaf nutrition content. By counting the number of significant correlation coefficients to identify sensitive bands for each combination, we identified 22 bands for leaf N, P, and K content.

### Development of new hyperspectral indices

We used stepwise linear regression to distinguish the optimal combination among the 22 sensitive bands selected earlier to identify the best combinations for estimating leaf nutrition for all indices. Following this, previously established methods were applied to devise high accuracy regression equations for leaf N, P, and K content^39^. The most sensitive bands finally selected for leaf N content were 468, 623, 624, 633, 652, 657, 668, 818, 821, 842, 937, and 938 nm (Table 1), which mostly lie in the red and NIR regions of the spectrum. The most sensitive bands for leaf P content were 416, 421, 424, 427, 458, 485, 664, 819, 828, 839, 902, and 933 nm, which lie in the visible green and NIR regions, and the most sensitive bands for K were 457, 483, 646, 731, 835, 900, 916, and 919 nm in the green, red, and NIR regions.

**Table 1:** Stepwise linear regression equations for leaf nutrition parameters, based on the sensitive bands selected from the reflectance and FD combinations.

Regression equations	R^2^	Adjusted R^2^
N = 21.6 − 2186.079D_937_ + 11429.702D_818_ − 1249.616D_938_ + 22063.134D_623_	0.762	0.712
P = 190.487 + 91712.256D_424_ − 54522.874D_416_ − 148213.752D_458_ + 7056.261D_902_	0.762	0.711
K = 61.532 + 12691.028D_919_ + 41079.969D_483_ + 37268.512D_457_	0.793	0.749
N = −6.087 − 4782.424ND_657_ − 7728.328ND_633_ + 3661.663ND_652_	0.776	0.729
P = 181.13 + 689.047ND_424_ + 55951.851ND_828_ − 4876.394ND_427_	0.717	0.679
K = 61.47 + 9721.848ND_919_ + 3614.272ND_457_ + 2891.555ND_483_	0.855	0.824
N = 4.874 − 69663.699FD_624_ + 197983.562FD_668_ + 28017.123FD_842_	0.876	0.850
P = 131.451 − 196845.744FD_839_ + 46413.765FD_819_ + 25813.846FD_421_	0.810	0.769
K = 105.882 − 64164.877FD_900_ − 43285.081FD_646_ − 26455.909FD_916_	0.795	0.751
N = 11.575 − 36198.245FDD_821_ + 38033.1FDD_468_ − 26486.514FDD_657_	0.879	0.854
P = 142.191 − 132036.035FDD_485_ + 288047.809FDD_664_ − 71641.791FDD_933_	0.838	0.803
K = 98.683 + 83049.321FDD_835_ + 64569.839FDD_731_	0.659	0.631

Note: D = Dij, FDD = FD(D), D_937_ indicates the D value at 937 band.

### Assessment of empirical hyperspectral indices

Forty-three empirical indices reported in previous publications were selected for identification of optimized indices (Table 2). Different degradation intensities demonstrated obvious variability in correlation coefficients. As reported in Peng *et al*.^39^, under light degradation, the values of Viopt, FD525–570, MSS-DVI, SDb, and SDr were significantly negatively correlated with measured contents. However, in severe degradation, the relations are significantly positive. Contrastingly, NVI and SDy demonstrated significantly positive relationships with light degradation and negative relations for severe degradation. Spectral indices for leaf nutrition contents perform differently according to degradation intensity.

**Table 2:** The correlation matrix of empirical indices with plant nutrition contents along degradation gradient.

Indices	Light degradation			Moderate degradation			Severe degradation		
	N	P	K	N	P	K	N	P	K
NDVI705	0.049	−0.204	−0.361**	0.003	−0.019	−0.226*	0.055	0.074	0.020
mNDVI705	0.096	−0.048	−0.215	0.113	−0.044	−0.216*	−0.034	0.083	−0.012
mSR705	0.170	−0.055	−0.213	0.077	−0.058	−0.226*	0.004	0.093	0.005
REP	0.262*	0.091	0.058	0.058	−0.005	−0.084	−0.143	0.065	−0.088
VOG1	0.206	−0.095	−0.223*	−0.008	−0.008	−0.198*	−0.058	0.057	0.015
VOG2	−0.298**	0.006	0.109	0.028	−0.005	0.137	0.067	0.000	0.039
VOG3	−0.288**	0.016	0.126	0.027	0.002	0.142	0.053	−0.008	0.033
PRI	0.161	0.046	−0.184	−0.023	−0.158	−0.181	0.120	0.054	0.213
OSAVI	−0.117	−0.327**	−0.522**	−0.011	−0.006	−0.190	0.090	0.083	0.033
NVI	0.419**	0.271*	0.214	0.024	−0.015	−0.002	−0.221*	−0.133	−0.268*
NDCI	0.053	−0.272*	−0.407**	−0.061	−0.006	−0.207*	0.066	0.008	−0.128
RI1dB	0.171	−0.112	−0.254*	−0.024	−0.015	−0.211*	−0.056	0.070	0.020
MCARI1	−0.044	−0.249*	−0.203	−0.276**	−0.141	−0.267**	0.267*	0.133	0.090
**DVI**	**−0.027**	−0.242*	**−0.178**	−0.223*	**−0.119**	−0.245*	**0.116**	**0.136**	**−0.006**
TVIBL	−0.144	−0.216	−0.109	−0.315**	−0.142	−0.247*	0.313**	0.127	0.111
GREEN-NDVI	0.096	−0.242*	−0.360**	−0.065	−0.022	−0.207*	0.009	−0.003	−0.129
Viopt	−0.020	−0.293**	−0.370**	−0.250*	−0.109	−0.204*	0.337**	0.154	0.122
RVI(810,560)	0.167	−0.187	−0.308**	−0.081	−0.044	−0.168	0.073	−0.031	−0.130
RVI(950,660)	0.019	−0.282*	−0.471**	−0.089	−0.029	−0.148	0.197	0.125	0.063
RVI(810,660)	0.006	−0.279*	−0.459**	−0.074	−0.030	−0.156	0.177	0.110	0.081
NDVI(573,440)	−0.275*	−0.485**	−0.439**	−0.094	0.057	0.019	0.123	0.114	0.058
FD730-525	−0.057	−0.181	−0.332**	−0.003	−0.017	−0.333**	0.192	−0.024	−0.032
FD730/525	0.269*	−0.079	−0.107	0.114	−0.066	−0.197*	0.011	−0.132	−0.165
FD(730-525)/(730 + 525)	0.195	−0.139	−0.148	0.147	−0.040	−0.082	−0.087	−0.001	−0.098
**FD730-570**	−0.230*	−0.233*	−0.420**	**−0.009**	**−0.035**	−0.313**	**−0.216**	**−0.056**	**−0.054**
FD730/570	−0.102	0.035	0.226	0.158	−0.101	0.340**	−0.333**	0.030	0.004
FD(730-570)/(730 + 570)	−0.059	0.120	0.060	0.191	−0.130	0.200*	0.065	−0.044	0.139
FD525-570	−0.502**	−0.188	−0.325**	−0.017	−0.058	−0.094	0.194	0.130	0.135
FD525/570	0.371**	0.098	0.167	−0.020	−0.046	−0.028	−0.158	−0.024	−0.082
FD(525–570)/(525 + 570)	−0.089	−0.183	−0.161	−0.258	0.020	0.056	0.182	0.191	0.141
MSS-DVI	−0.380**	−0.333**	−0.488**	−0.262**	0.002	−0.384**	0.259*	0.093	0.117
MSS-PVI	0.007	−0.213	−0.143	−0.137	−0.073	−0.188	−0.009	0.113	−0.065
MSS-SARVI	−0.012	−0.333**	−0.500**	−0.084	−0.016	−0.130	0.232*	0.058	0.040
AVHRR-GVI	−0.152	0.053	0.250*	−0.119	−0.040	0.024	0.157	−0.071	0.084
SDr-SDb	−0.357**	−0.255*	−0.411**	0.035	−0.016	−0.331**	0.229*	0.120	0.067
**RES**	−0.370**	−0.300**	−0.412**	**−0.066**	**−0.033**	−0.363**	**−0.212**	**−0.119**	**−0.116**
SDb	−0.510**	−0.179	−0.239*	−0.123	−0.013	−0.123	0.196	0.152	0.144
Sdy	0.357**	0.355**	0.487**	−0.272**	−0.045	0.059	−0.196	−0.149	−0.164
SDr	−0.409**	−0.264*	−0.417**	0.020	−0.017	−0.329**	0.228*	0.128	0.081
SDr/SDb	0.214	−0.034	−0.145	0.094	0.022	−0.194*	−0.080	0.023	−0.036
SDr/SDy	0.025	0.150	−0.092	0.172	−0.079	0.038	−0.074	−0.123	−0.182
(SDr-SDb)/(SDr+SDb)	0.072	−0.071	−0.203	0.167	−0.003	−0.232*	0.087	0.049	−0.063
(SDr-SDy)/ (SDr + SDy)	−0.251*	−0.244*	−0.353**	0.250*	0.012	0.023	0.131	0.096	0.080

The bold indices indicate the selected indices, which have steady and significant correlation coefficients with plant nutrition contents over all degradation gradients.

Note: The significant level is indicated by * (at 0.05 level) or ** (at 0.01 level).

We selected three spectral indices for leaf nutrition estimation (RES, DVI, and FD730-570) for their ease of use and accuracy. These indices have high (Table 2). A comparison of R^2^ values between the optimized stepwise regression indices derived from the complete combinations (Table 1) and the empirical indices (Table 2) showed that the R^2^ values of the proposed stepwise regression indices were significantly higher than the best performing empirical indices.

We then aimed to establish a suitable equation for each of the three selected indices from Table 2. Linear regression equations were constructed (Table 3) based on field measured leaf N, P, and K contents and the corresponding empirical indices RES, DVI, and FD730-570 for all three degradation intensities. These indicated that all three empirical indices can predict leaf nutrition content at a statistically significant level, with the exception of RES and DVI for leaf P content.

**Table 3:** The model predictions for leaf nutrition parameters from the empirical spectral indices selected in Table 2.

Indices	N	R^2^	P	R^2^	K	R^2^
RES	y = −786.75x + 13.733	R² = 0.1373**	y = −6221.3x + 146.95	R² = 0.09	y = −7846.6x + 131.48	R² = 0.169**
DVI	y = −0.0017x + 32.711	R² = 0.050*	y = −0.0024x + 181.27	R² = 0.014	y = −0.0028x + 95.466	R² = 0.06*
FD730-570	y = −440.44x + 10.251	R² = 0.053*	y = −4351.8x + 126.87	R² = 0.054*	y = −7213.4x + 120.97	R² = 0.177**

Note: The significant level is 0.05 level (*) or 0.01 level (**).

### Validating selected empirical and newly developed hyperspectral indices

**Figure 4:**
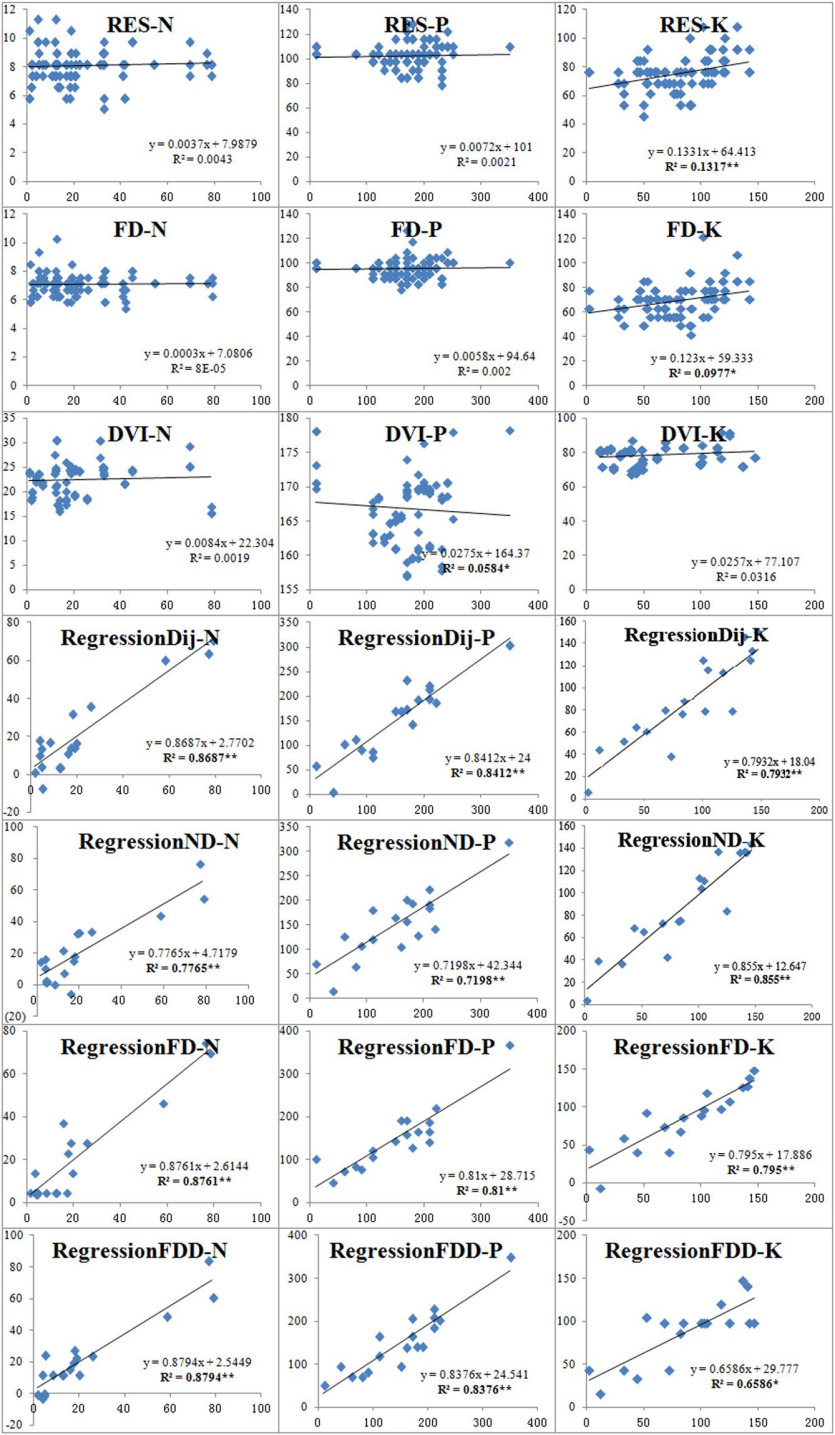
Linear regression for field survey (X axis) and predicted (Y axis) values of leaf N, P, and K contents (mg/g) based on newly developed and empirical spectral indices across various degraded vegetation areas in Helin County, China. Determining coefficients in bold (R^2^) indicate significance at 0.05 (*) or 0.01 (**).

Referencing Peng *et al*. 2018, we used the newly developed (Table 1) and published empirical (Table 3) spectral indices for estimating plant leaf nutrition content on validation samples. Linear regression and correlation coefficients of the predicted values accurately reflected leaf nutrition contents in the field measurements at a statistically significant level (Fig. 4). Confirming previsouly published results, the R^2^ of the empirical indices predictions was lower than the one calculated from the newly developed indices, both the new and empirical hypersspectral indices predicted leaf K content better leaf N and P content.

## Discussion

A handheld spectrometer directly acquires detailed spectra located in the visible and near-infrared regions bands related to leaf N, P, and K^22,30^. Many empirical spectral indices have been developed based on correlation analysis of field survey and satellite remote sensing data, including the 43 indices used in this study. However, retrieving leaf nutrition information from satellite remote sensing data and space-based observations is challenging because of the influence of atmospheric effects. Vegetation characteristics and background reflectance may also confound the compound signals received by the remote sensors^40^. Therefore, leaf nutrition status can be better simulated by spectral indices based on narrow and sensitive bands which experience less atmospheric influence and background disturbance, for both multispectral satellites and hyperspectral spectrometers. Based on this hypothesis, we combined the reflectance and its first-order derivative value at every waveband and sensitive ranges acquired by the ASD spectrometer. Bands sensitive to leaf nutrition content were identified and the most suitable equations of combined narrative bands were selected and applied to predict leaf nutrition content. The results demonstrate that plant leaf N, P, and K contents can be better predicted by the newly developed models than by solely empirical spectral indices.

The utilization of FD values contributes to the high accuracy of the results. Obtaining FD spectra by the division of difference in reflectance between successive wavebands eliminates the overlapping spectral features and background noise^41^. FD is currently used to decompose a mixed spectrum and reduce the noise in the hyperspectral region^17^. Spectral indices based on FD are found to be highly sensitive to many of the physiological parameters of leaves, and are therefore strong predictors of leaf nutrition content^42,43^. However, few studies have examined the performance of FD spectra across the 400–900 nm wavelengths. Our study did so and produced a complete combination of FDs. The usage of FD improves the performance of our proposed hyperspectral indices. Stepwise linear regression analysis of FD in our study may also greatly improve the estimation of leaf nutrition status, by avoiding potential overfitting problems when the number of variables is considerably fewer than the number of samples^44^. When the number of variables is limited, potential confounding factors are preferable to employing a simple index-based approach.

The best results were also attributed to the use of sensitive band identification in hyperspectral data. Although selecting sensitive bands is extremely important for increasing the accuracy of estimation, the method for carrying out this selection is a challenging issue. Wavelengths identified as most sensitive to N vary between studies. Zhao *et al*. (2005) found that leaf N was most responsive at 517 and 701 nm in cotton^9^, while Buscaglia and Varco (2012) identified a stronger relationship between cotton leaf N content and leaf or canopy reflectance in the green wavelengths instead of the red-edge or NIR region^14^. Using six different models, Yao *et al*. (2015) found that 690/695, 709/710, 700/705, 713/727, 1200, and 1335/1340 nm, located in the red-edge and near-infrared regions, were the sensitive wavelengths for N^17^.

However, for leaf N estimation in wheat, wavelengths of 384, 492, 695, 1339, and 508 nm and 681, 722, 960, 1264, and 1369 nm were found to perform best^17^. Among these bands, chlorophyll and carotenoids in green plants often strongly absorb the visible range 384, 492, and 508 nm; 681, 695, and 722 nm in the red range and can serves as sensitive N indicators; and the shortwave infrared range 960, 1264, 1339, and 1369 nm bands are indicators of proteins (where N is a main component)^39^. In our study, the sensitive bands for leaf N content from temperate degraded vegetation were found to be 468, 623, 624, 633, 652, 657, 668, 818, 821, 842, 937, and 938 nm, in the red and NIR regions.

Various factors can affect the accuracy of leaf N estimates. Tarpley *et al*. (2000) found that leaf N can be overestimated by indices constructed from green or yellow-orange wavelengths, potentially due such confounding factors as macro and micronutrient deficiencies^45^. When comparing the active and passive sensors used to discriminate nitrogen status, Erdle *et al*. (2011) found a saturation effect with the increase in leaf N content^46^, possibly induced by changing photosynthetic photon flux density affecting several pigments. In addition, the measurement scale can affect the sensitive band selection. Read *et al*. (2002) reported that wavelengths sensitive to leaf N content also shifted from 405, 585, 695, 755, 845, and 925 nm at the leaf scale to 410, 605, and 700 nm at the canopy scale^47^, and that the bands suitable for estimating leaf N varied between different plant growth stages. Buscaglia and Varco (2012) found that the wavelength most sensitive to leaf N, and consequently best correlated with cotton leaf N content, was 612 nm at squaring and 728 nm at the flowering stage^14^.

The newly developed spectral models, based on sensitive bands and optimized by combination, have high determinant coefficients under various environmental conditions. The three degradation intensity environments included various conditions, which can affect the results. First, an increase in leaf N content from severely degraded vegetation to lightly degraded vegetation may induce a saturation effect. Furthermore, deficiencies in macro and micro-nutrients in severely degraded vegetation under stress conditions may induce an overestimation of leaf N content. Finally, different development stages among dominant plant species may induce a shift in the sensitive bands of leaf N content. Under these conditions and many possible disturbance factors, the newly developed spectral models are relatively steady and robust in their estimations of leaf N content.

The same process of hyperspectral data analysis was used to estimate leaf P and K content and also yielded the best results. The identification of narrower sensitive bands was achieved by comparing the correlation coefficients for combinations of indices. Stepwise linear regression analysis was then conducted on these sensitive bands for each of the three degradation intensities. This can increase the accuracy of leaf P and K estimation by considering environmental conditions across various species. The sensitive bands for P were determined to be 416, 421, 424, 427, 458, 485, 664, 819, 828, 839, 902, and 933 nm, in the visible green and NIR regions of the spectrum. The sensitive bands for K were found to be 457, 483, 646, 731, 835, 900, 916, and 919 nm, which lie in the green, red and NIR regions. These bands are located within ranges reported in previous studies^22,30^, and show higher correlation coefficients. Since these bands were extracted from six dominant species in temperate vegetation, they can be applied more generally.

The high accuracy of newly developed spectral models may be attributable to the deletion of the spectral water absorption region, which was done at the start of hyperspectral data processing. Water absorption mainly affects spectra above 790 nm^8^. We used FD and combinations of different sensitive bands to weaken such effects. The utilization of hyperspectral data with hundreds of bands may also help to increase the accuracy of leaf nutrition status estimates. Previous studies show no shared optimal three-band spectral index. Instead, a normalized difference spectral index can be utilized to estimate leaf N, P, or K content in different plant species^8,48^. Obviously, it is more precise to make estimates by selecting several sensitive bands from the hundreds available than to only use several fixed bands. Most satellite remote sensing data have only four to seven bands, which limits their ability to estimate the physiological parameters of leaf health.

The new spectral models were developed with general applicability in mind. First, the models extracted spectral information from six dominant species, including woody plants, shrubs, and grasses, representing wide spectral characteristics of various species. Second, we tested the accuracy of models across various degradation intensities, and only models performing with high consistency across various vegetation states were selected. Third, the complete combination of original reflectance and its first-order derivative values over 350–1075 nm, the wavelength mostly used by majority of spectrometers, have wide potential for application. With these considerations, these new models may help to monitor degraded vegetation. However, analysis must be mindful of the dominant species in vegetation across ecosystems when using our developed spectral models to estimate leaf nutrition contents, since different species demonstrate different spectral traits even when they have the same chemical contents. In addition, more advanced methods such as partial least squares regression, support vector regression, and random forest are increasingly used for analyzing hyperspectral data^49,50^, which can also predict leaf nutrition contents and are therefore strongly suggested in future study.

## Conclusions

This study used completed combinations of sensitive bands and 43 empirical spectral indices from three datasets collected *in-situ* from lightly, moderately, and severely degraded vegetation in temperate Inner Mongolia, China to estimate leaf nutrition contents. Among empirical indices, RES, DVI, and FD730-570 performed best. Stepwise linear regression on reflectance difference (Dij), normalized differences (ND), first-order derivative (FD), and first-order derivative difference (FD(D)) at sensitive bands were selected using Pearson correlation analysis under various community conditions and were found to be the most effective in predicting leaf nutrition contents (R^2^ = 0.5–0.8, p < 0.05). These indices, extracted from narrow sensitive bands, were statistically significant and performed better than empirical indices. Therefore, they can be regarded as a global index which sufficiently represents nutritional content. This demonstrates great potential for the use of hyperspectral data in monitoring leaf nutrition status at a fine scale. These spectrally very narrow models can only be applied with very high spectral resolution of 1–3 nm spectrometer. However, curves of the correlation coefficient of determination can aid in locating indices tailored to other remote sensors. This new understanding may help to explore the potential for hyperspectral data in quantifying leaf nutrition content. In addition, it would be useful to test the proposed indices using image aerial and satellite hyperspectral data in future studies, to provide a set of indicators with wider generality.
